# How Oxygen Availability Affects the Antimicrobial Efficacy of Host Defense Peptides: Lessons Learned from Studying the Copper-Binding Peptides Piscidins 1 and 3

**DOI:** 10.3390/ijms20215289

**Published:** 2019-10-24

**Authors:** Adenrele Oludiran, David S. Courson, Malia D. Stuart, Anwar R. Radwan, John C. Poutsma, Myriam L. Cotten, Erin B. Purcell

**Affiliations:** 1 Department of Chemistry and Biochemistry, Old Dominion University, Norfolk, VA 23529, USA; aolud001@odu.edu (A.O.); dcourson@odu.edu (D.S.C.); 2 Biology Department, Palomar College, San Marcos, CA 92069, USA; maliastuart5@gmail.com; 3 Department of Chemistry, College of William and Mary, Williamsburg, VA 23185, USA; aradwan@email.wm.edu (A.R.R.); jcpout@wm.edu (J.C.P.); 4 Department of Applied Science, College of William and Mary, Williamsburg, VA 23185, USA

**Keywords:** host defense peptides, membrane activity, copper, piscidins, *Clostridioides difficile*

## Abstract

The development of new therapeutic options against *Clostridioides difficile* (*C. difficile*) infection is a critical public health concern, as the causative bacterium is highly resistant to multiple classes of antibiotics. Antimicrobial host-defense peptides (HDPs) are highly effective at simultaneously modulating the immune system function and directly killing bacteria through membrane disruption and oxidative damage. The copper-binding HDPs piscidin 1 and piscidin 3 have previously shown potent antimicrobial activity against a number of Gram-negative and Gram-positive bacterial species but have never been investigated in an anaerobic environment. Synergy between piscidins and metal ions increases bacterial killing aerobically. Here, we performed growth inhibition and time-kill assays against *C. difficile* showing that both piscidins suppress proliferation of *C. difficile* by killing bacterial cells. Microscopy experiments show that the peptides accumulate at sites of membrane curvature. We find that both piscidins are effective against epidemic *C. difficile* strains that are highly resistant to other stresses. Notably, copper does not enhance piscidin activity against *C. difficile.* Thus, while antimicrobial activity of piscidin peptides is conserved in aerobic and anaerobic settings, the peptide–copper interaction depends on environmental oxygen to achieve its maximum potency. The development of pharmaceuticals from HDPs such as piscidin will necessitate consideration of oxygen levels in the targeted tissue.

## 1. Introduction

*Clostridioides* (formerly *Clostridium*) *difficile* infection (CDI), whose symptoms can include inflammation, profuse diarrhea, and pseudomembranous colitis, has been recognized as an urgent public health threat in the United States and other industrialized nations [1,2]. CDI is primarily a hospital-acquired disease, as disruption of the native gut microbiota by prior antibiotic usage is the major risk factor for *C. difficile* colonization, although the number of community-acquired infections has increased in recent years [3,4]. The severity of CDI has also increased during the 21st century with the emergence of so-called “hypervirulent” epidemic ribotypes of the bacterium, most notably ribotype 027, that are associated with higher levels of disease recurrence and death in infected patients [3,5,6,7]. *C. difficile* is resistant to several families of antibiotics, including penicillin-family beta lactams and fluoroquinolones, and is increasingly resistant to next-generation therapeutics including fidaxomicin and vancomycin [6,8,9]. Currently, the most clinically effective treatment for CDI is replenishment of the protective gut microbiota through fecal transplants. As these procedures have a high inherent risk of introducing uncharacterized pathogens and are not recommended for immunocompromised patients, there is great interest in the development of new strategies for prevention and treatment [10,11,12,13].

*C. difficile* persists in the environment in the form of metabolically dormant spores, which are highly resilient to chemical and physical stresses and remain viable for months [14,15]. If mammals ingest these spores, amino acids and bile salts in the digestive system trigger their germination into metabolically active vegetative cells [16,17,18]. Vegetative *C. difficile* often cannot integrate well into the diverse, metabolically efficient microbial ecosystem of a healthy intestinal microbiome but can take advantage of the loss of bacterial species diversity and rise in nutrient availability induced by antibiotic exposure to establish colonization [19,20,21,22]. *C. difficile* colonization triggers the innate immune response, including the release of reactive oxygen species (ROS) and cationic host defense peptides (HDPs) [23,24,25]. These antimicrobial peptides can kill bacterial cells directly through a number of mechanisms, attacking the cell membrane and/or intracellular targets, and indirectly by activating the host innate immune response [26,27,28,29]. As these peptides have multiple cellular targets, bacteria cannot quickly develop or transmit genetically encoded resistance to them, and they are a promising precursor for the development of stand-alone antibiotics or adjuvants designed to work synergistically with existing antibiotics [30,31].

Piscidins are a family of HDPs found in teleost (bony) fish species with demonstrated efficacy against a wide range of bacteria and viruses [32,33,34,35]. The piscidins p1 (FFHHIFRGIVHVGKTIHRLVTG) and p3 (FIHHIFRGIVHAGRSIGRFLTG), which are derived from the mast cells of hybrid striped sea bass, exhibit broad spectrum antibacterial activity although their mechanisms of action differ [36,37]. Both peptides localize to bacterial cell membranes and are internalized at sub-lethal concentrations. While p1 is more damaging to membrane integrity than p3, the latter is more disruptive to DNA [36]. Furthermore, studies done on live bacteria and model membranes indicate that the peptides, especially p1, take advantage of lipid heterogeneity to deploy their mechanism of membrane disruption [36,38]. Recently, we demonstrated that under aerobic conditions both peptides use their amino-terminal copper- and nickel-binding (ATCUN) motifs to coordinate Cu^2+^ with picomolar affinity [37,39]. Piscidin-copper complexes form ROS and exhibit nuclease activity against double stranded DNA, resulting in increased lethality against multiple bacterial species [37]. Such copper-ATCUN complexes can serve as sources of oxidative stress, increasing peptide lethality against bacteria in an aerobic environment [40,41,42]. Oxidative stress can be harmful or lethal to organisms, depending on their oxidative stress tolerance. Obligate anaerobes such as *Clostridia* are considered completely intolerant to oxygen, although they can employ scavenger and reductase enzymes to survive transient exposure to environmental oxygen or immune-mediated oxidative bursts [15,23,43,44,45]. Application of antimicrobial peptides sensitizes *C. difficile* to antibiotics, although epidemic strains from ribotype 027 are less sensitive than other strains [31,46].

Importantly, as HDPs, piscidins have immunomodulatory effects. In particular, our investigations have demonstrated that both p1 and p3 induce chemotaxis in neutrophils [39]. These effects are exclusively mediated by formyl peptide receptors 1 and 2 (FPR1 and FPR2), both of which are G-protein coupled receptors (GPCRs) that play important functions in the immune system [47,48,49,50,51]. Interestingly, Cu^2+^-coordination decreases the chemotactic effects of p1 and p3, suggesting a regulatory effect of copper between the direct and indirect antimicrobial effects of the peptides [39]. Given the role of FPR2 for the resolution of inflammation, it has become an important drug target [47,48,49]. P1 has also been shown to decrease the inflammatory response through a process that may involve binding lipopolysaccharides and decreasing toll-like receptor (TLR)-mediated inflammatory pathways [52,53]. The immunomodulatory properties of HPDs such as piscidin have emerged as an important topic of research given that these effects are indirect, and thus unlikely to activate mechanisms of drug resistance observed with traditional antibiotics that directly attack bacteria [54,55,56,57,58,59]. In addition, HDP modulation of the inflammatory immune response can mitigate infection symptoms and has been shown to reduce toxin-dependent inflammation in mouse models of *C. difficile* infection [60].

As indicated above, the antimicrobial effects of piscidin have previously been measured in aerobic environments. However, there is a ten-fold range of partial oxygen pressure among the tissues of the human body, with organs such as the large intestine providing a habitat for anaerobic microbes, both commensal and pathogenic [61]. Here, we report that the antimicrobial activity of p1 and p3 differ in aerobic and anaerobic environments. In an anaerobic environment, both p1 and p3 are incorporated into *C. difficile* cells, inhibit bacterial proliferation, and are highly toxic against actively dividing *C. difficile*. Both peptides associate extensively with bacterial cell membranes, exhibiting preferential localization at sites of high curvature such as cell poles and septa. In contrast to previously observed aerobic data, anaerobic piscidin antibacterial activity does not appear to be enhanced by metal complex formation. Our findings suggest that the mechanism by which these peptides induce bacterial cell death is influenced by the availability of environmental oxygen. It is clear that future mechanistic investigations of HDPs focused on potential medical applications must account for oxygen levels at the desired site of action in order to accurately model antimicrobial activity.

## 2. Results

### 2.1. Piscidins Are Incorporated into C. difficile and Appear to Localize to Sites of Membrane Curvature

**Figure 1:**
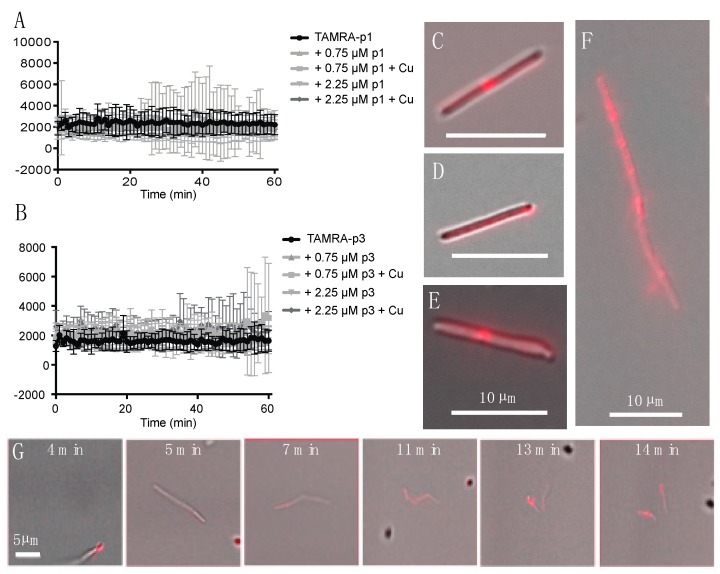
Incorporation of TAMRA-piscidin into live *C. difficile.* (**A**,**B**) Fluorescent signal per cell of 0.75 μM TAMRA-labeled p1 (**A**) and p3 (**B**) mixed with live *C. difficile* R20291. Cells had already reached maximum peptide incorporation by the time recording began, roughly 6 min after peptides and cells were mixed. Addition of unlabeled peptide, in the presence or absence of equimolar amounts of copper sulfate, did not cooperatively increase peptide incorporation. (**C**–**F**) Representative images of *C. difficile* labeled with: (**C**) 0.75 μM TAMRA-labeled p1 plus 0.75 μM unlabeled p1; (**D**) 0.75 μM TAMRA-labeled p3; (**E**) 0.75 μM TAMRA-labeled p1 plus 2.25 μM unlabeled p1; (**F**) 0.75 μM TAMRA-labeled p1 plus 0.75 μM unlabeled p1. (**G**) Timecourse showing the rupture of a pre-divisional cell labeled with 0.75 μM TAMRA-labeled p1 plus 0.75 μM unlabeled p1. Scale bars in panels (**C**–**F**) represent 10 μm. Scale bar in panel (**G**) represents 5 μm.

Confocal microscopy of fixed bacterial cells exposed to fluorescently labeled p1 and p3 has previously shown that they enter both Gram-negative and Gram-positive bacterial cells and appear to be concentrated at bacterial nucleoids and cell septa [36,39]. We exposed live *C. difficile* R20291 cells to 0.75 μM 5-carboxytetramethylrhodamine (TAMRA)-labeled p1 and p3 and observed peptide uptake and localization in unfixed live cells. As *C. difficile* exhibits green autoflouresence, the red TAMRA labeling was distinct from any intrinsic signal produced by the cells [62]. Exponential-phase cells and peptides were mixed and sealed within microscopy chambers in an anaerobic chamber and then transported to the microscope, resulting in a 6-min delay between the onset of peptide exposure and the first image [63]. Mean fluorescence intensity within cells was stable over the course of 1 h of monitoring, indicating that peptide incorporation into cells occurs within the first few min of exposure (Figure 1A,B). Peptide integration appeared to be complete within 6 min even at lower peptide concentrations of 0.25 and 0.075 μM (data not shown). The addition of additional unlabeled peptide or unlabeled peptide complexed with Cu^2+^ did not increase fluorescence intensity, and thus there was no evidence of potential cooperativity in peptide uptake. There were distinct fluorescent puncta at the septa of predivisional cells (Figure 1C,E,F), consistent with prior observations in *Escherichia coli* (*E. coli*) and *Bacillus megaterium* (*B. megaterium*) [36]. In addition, there were fluorescent puncta at cell poles, suggesting that piscidins generally localize to sites of high curvature (Figure 1D). While unlabeled cells were motile and maintained rod-like shapes, many of the fluorescently labeled cells exhibited curvature or surface irregularities suggestive of cell envelope damage (Figure 1E). Performing these experiments on live cells allowed real-time observation of cellular response to peptide intoxication. We observed a motile chain of predivisional rod-shaped cells over the course of 10 min (Figure 1G). During this time, the chain of cells took up labeled p1 at one pole and subsequently developed progressively severe curvature at cell septa and separated into smaller fragments (Figure 1G). The resulting pieces were asymmetrically curved and non-motile, indicating that lysis rather than healthy cell division had occurred.

### 2.2. Piscidins Prevent C. difficile Proliferation

**Figure 2:**
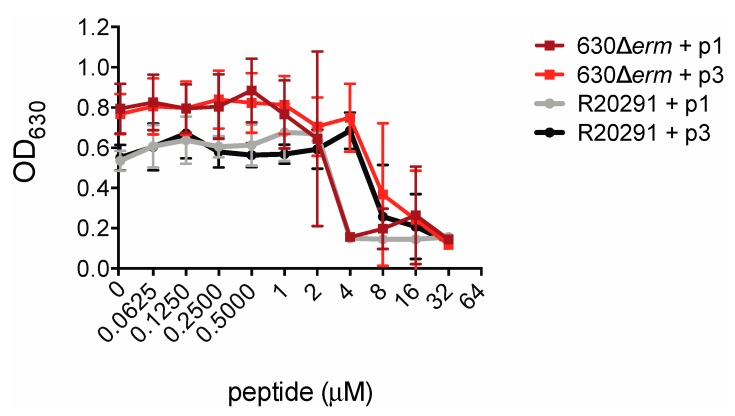
Piscidins inhibit *C. difficile* growth. Optical densities of overnight *C. difficile* 630Δ*erm* and R20291 cultures grown in the presence of the indicated concentrations of piscidins. Data shown are the means and standard deviations of four biologically independent samples.

In order to measure the inhibitory effects of piscidin peptides on *C. difficile* growth we inoculated *C. difficile* strains 630Δ*erm* and R20291 into a medium containing the peptides. *C. difficile* 630Δ*erm* is an erythromycin-sensitive derivative of the reference strain *C. difficile* 630, while R20291 is an epidemic strain isolated from a 2003–2005 hospital outbreak of *C. difficile* infection in the United Kingdom [64,65]. R20291 is a so-called “hypervirulent” strain of ribotype 027 and is more resistant than 630 to multiple classes of antibiotics including clindamycin and ciprofloxacin [66,67,68,69]. The presence of piscidin peptides prevented *C. difficile* proliferation. Notably, R20291 was as susceptible as 630Δ*erm* to growth inhibition by piscidin. p1 inhibited proliferation of both strains at concentrations at or above 4.00 μM and p3 inhibited growth at or above 8.00 μM (Figure 2).

### 2.3. Piscidins Reduce Established C. difficile Populations

**Figure 3:**
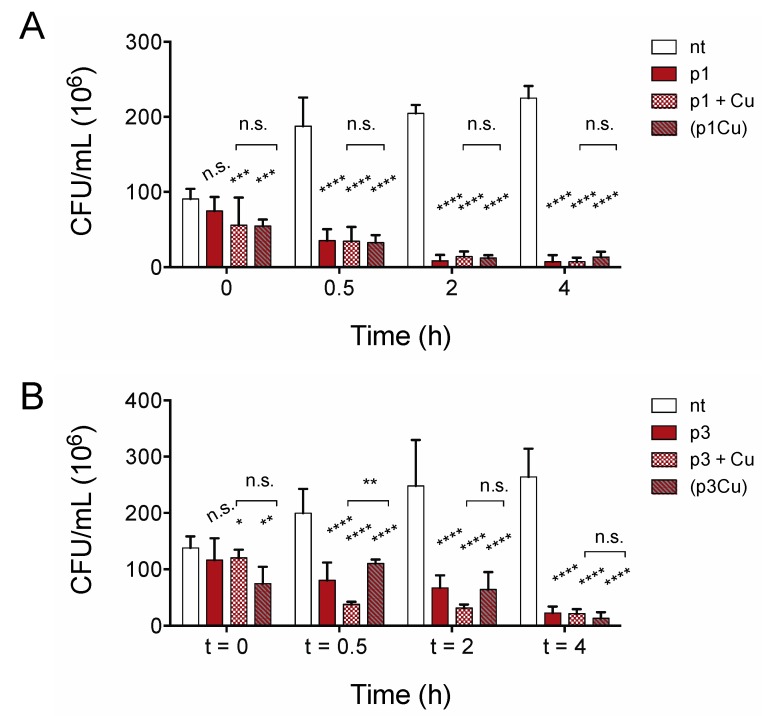
Copper does not accelerate anaerobic *C. difficile* killing by piscidins. Time-kill assays comparing viable colony forming units per milliliter (CFU/mL) of bacterial culture before exposure to p1 (**A**) and p3 (**B**) with the CFU/mL 30 min, 2, and 4 h post-exposure. Cells were exposed to peptides (p1 and p3), peptides and equimolar copper sulfate added simultaneously (p1 + Cu and p3 + Cu), and peptides allowed to form piscidin-copper complexes in an aerobic environment prior to addition to the anaerobic bacterial cultures ((p1Cu) and (p3Cu)). CFU/mL in treated samples were compared to those in untreated samples and to each other using two-way ANOVA with Tukey’s post-test comparison. nt, not treated; n.s., not significant; * *p* < 0.05; ** *p* < 0.01; *** *p* < 0.001; **** *p* < 0.0001.

Growth inhibition assays do not distinguish between substances that kill cells and bacteriostatic substances that inhibit growth only if compounds are present in sufficient quantities prior to bacterial proliferation. To confirm that piscidins are capable of reducing the number of viable cells in established bacterial populations, we performed time-kill assays to confirm that the number of viable *C. difficile* R20291 cells in exponentially growing culture decreases with exposure to p1 and p3 at sub-inhibitory concentrations. As shown in Figure 3, both p1 and p3 reduce *C. difficile* viability at half of the concentration needed to inhibit bacterial growth. The addition of 2.00 μM p1 significantly reduces the number of viable cells in the culture within 30 min, with continued loss of colony forming units over the course of 4 h (Figure 3A). Similarly, incubation with 4.00 μM p3 significantly reduced the number of viable cells within 30 min (Figure 3B). The bacterial killing by both p1 and p3 against *E. coli* in aerobic environments is exacerbated by the addition of equimolar copper, which complexes with the peptides, resulting in covalent damage to lipids and DNA [37]. We investigated the effect of adding copper sulfate to the anaerobic *C. difficile* killing assays at the same molar concentration as the piscidins. The addition of copper ions had no observable effect on p1 lethality at any of the timepoints examined (Figure 3A). Copper did appear to accelerate killing by p3 at 30 min and 2 h post-treatment, but the differences between peptide alone and peptide with copper had disappeared by 4 h post-treatment (Figure 3B). To investigate the possibility that copper ions were being chelated by other factors present in the tryptone-yeast (TY) medium and not actually forming complexes with piscidins, we repeated the assays with pre-formed piscidin-copper complexes. Copper allowed to form complexes with p1 prior to addition to the bacteria cultures still had no additional impact on p1 killing of *C. difficile* (Figure 3A) compared to apo p1. Pre-formed p3-copper complexes behaved like p3 in the absence of copper, killing *C. difficile* more slowly than p3 with copper added separately, but differences between the two conditions disappeared within 4 h (Figure 3B). This confirms that copper does not enhance the antimicrobial effects of piscidins under anaerobic conditions.

### 2.4. Copper Is Toxic to Anaerobically Growing C. difficile

**Figure 4:**
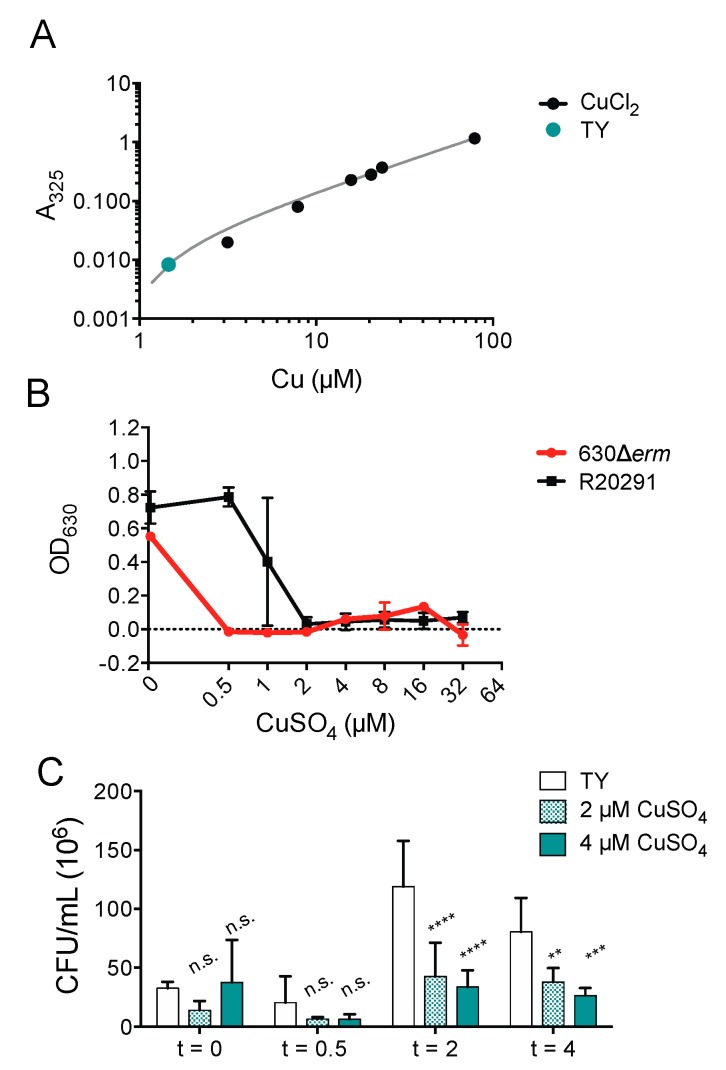
Copper is still antimicrobial in anaerobic environments. (**A**) Copper concentration of TY medium. (**B**) Optical densities of overnight *C. difficile* 630Δ*erm* and R20291 cultures grown in the presence of the indicated concentrations of copper sulfate. Data shown are the means and standard deviations of four biologically independent samples. (**C**) Time-kill assays comparing viable colony forming units per milliliter (CFU/mL) of bacterial culture before exposure to the indicated concentrations of copper sulfate with the CFU/mL 30 min, 2, and 4 h post-exposure. CFU/mL in treated samples were compared to those in untreated samples and to each other using two-way ANOVA with Tukey’s post-test comparison. nt, not treated; n.s., not significant; ** *p* < 0.01; *** *p* < 0.001; **** *p* < 0.0001.

It has been previously suggested the antimicrobial synergy of ATCUN-containing HDPs depends upon environmental dioxygen, which could account for the inability of copper ions to enhance piscidin activity against *C. difficile* [40,70,71]. To define the effects of copper alone against *C. difficile*, we determined that the copper concentration of the TY growth medium used to culture *C. difficile* under anaerobic conditions (Figure 4A) is 1.46 μM, which is lower than that of the Mueller-Hinton broth previously used to culture *E. coli* and *Pseudomonas aeruginosa* under aerobic conditions [37,72]. We found that copper salts are capable of inhibiting *C. difficile* growth at the same concentrations used in the time-kill assays with piscidins. The epidemic strain R20291 is less susceptible to copper inhibition that the historical strain 630Δ*erm* (Figure 4B). Actively dividing R20291 cells in the exponential phase are also killed by exposure to micromolar concentrations of copper sulfate (Figure 4C).

## 3. Discussion

The development of new therapies to combat antibiotic-resistant infections, including *C. difficile* infection, is an urgent public health priority. Antimicrobial peptides capable of simultaneously killing bacterial pathogens and stimulating the host innate system are a promising avenue for the development of new antimicrobial therapies. Piscidins previously showed efficacy against Gram-negative and -positive bacteria in aerobic environments [37]. Here, we investigated their effect on anaerobic bacteria. While piscidins are still highly lethal against *C. difficile* growing anaerobically, we found that piscidin growth inhibition and killing against *C. difficile* were not enhanced by the addition of Cu^2+^. This is true despite the fact that copper alone is capable of inhibiting *C. difficile* growth and killing actively growing *C. difficile* cells. While in aerobic environments, the bacterial response to piscidins with Cu^2+^ is greater than that to either piscidins or copper alone, it appears that the anaerobic response to piscidin with copper is less than the sum of the parts, and thus does not feature the synergistic effects observed aerobically.

Bacteria employ general stress response pathways that can be activated by multiple diverse extracellular stresses. Bacterial cells that have previously been exposed to an extracellular stress, such as starvation or oxidative stress, demonstrate increased resilience against unrelated threats, such as antibiotic exposure [73,74]. The fact that the *C. difficile* strains 630Δ*erm* and R20291 exhibit identical inhibition in response to p1 and p3, but differential inhibition to copper alone, suggests that piscidins and copper inhibit bacterial growth and viability by different mechanisms. This makes sense because Cu^2+^ has no specificity while piscidin does. It should be noted that free Cu^2+^ in vivo is highly toxic to mammalian as well as bacterial cells, and complexation by HDPs such as p1 and p3 can provide the critical specificity of targeting the metal ions to bacterial rather than host cells. HDPs are a viable treatment option against both aerobic and anaerobic bacteria, and in the case of *C. difficile*, it is extremely encouraging that the epidemic strain R20291 is just as susceptible to HDP inhibition and killing as the less robust 630Δ*erm* strain. As the symptoms of *C. difficile* infection are largely inflammatory, and treatments based on piscidins could potentially reduce inflammation while killing the causative pathogen, this is a very promising strategy to pursue. However, it is clear that while clinical antibiotics derived from HDPs would benefit from the inclusion of copper in tissues with high levels of oxygen, such as the lungs, antibiotics targeted to less aerobic tissues, such as the kidneys or large intestine, may not. Future work to investigate the interaction of piscidins with *C. difficile* in animal models of infection will be necessary to determine whether copper could or should be included with the peptides. More broadly, it appears that clinical treatments developed from HDPs should be designed in a tissue-specific manner, as metal ion adjuvants may be beneficial or necessary in some organs and unneeded in others, based on the oxygen levels at the site of activity.

## 4. Materials and Methods

### 4.1. Materials, Chemicals, Bacterial Strains and Growth Conditions

Materials and chemicals were purchased from Fisher Scientific (Hampton, NH, USA) unless otherwise indicated. The bacterial strains used in this study are listed in Table S1. *C. difficile* 630Δ*erm* and R20291 were maintained on brain-heart infusion supplemented with 5% yeast extract (BHIS) agar plates and liquid cultures were grown in TY medium [16,75,76]. All anaerobic bacterial culture took place at 37 °C in a Coy anaerobic chamber (Coy Laboratory Products, Grass Lake, MI) with an atmosphere of 85% N_2_, 10% CO_2_, 5% H_2_. All plastic consumables were allowed to equilibrate in the anaerobic chamber for a minimum of 72 h.

### 4.2. Peptide Synthesis

Caboxyamidated p1 (MW 2571) and p3 (MW 2492) were synthesized using Fmoc chemistry at the University of Texas Southwestern Medical Center (Dallas, TX, USA). For the TAMRA-labeled forms of the peptides, the fluorescent label was attached to the amino-end of the peptides before cleavage from the resin. The peptides were purified at William and Mary on a Waters HPLC system using a C18 X-Bridge Waters column (Milford, MA, USA) and acetonitrile/water gradient acidified with 0.1% trifluoroacetic acid, as previously described [77,78]. After removal of the organic phase, the peptides were lyophilized. Next, they were dissolved in dilute HCl and dialyzed to remove residual trifluoroacetic acid. The purification steps yielded 98% pure peptides based on HPLC chromatograms and mass spectrometry data. HPLC chromatograms and mass spectra collected at William and Mary on the purified peptides are included in the Supplementary Information (Figures S1 and S2). The purified peptides were dissolved in nanopure water. The concentrations of p1 and p3 were determined by amino acid analysis at the Texas A&M Protein Chemistry Center (College Station, TX, USA). Metallation of each peptide was achieved when an equimolar of CuSO_4_ was added to the medium (see below). For the TAMRA-labeled peptides, the absorbance at 547 nm was used to quantify their concentrations. To avoid photobleaching of the fluorescent probe, the TAMRA-labeled peptides were protected from light by wrapping containers with foil.

### 4.3. Microscopy

Live-cell, time-lapse, wide-field fluorescence, and differential interference contrast (DC) microscopy of the interaction between TAMRA-labeled piscidin peptides and *C. difficile* R20291 bacteria was performed on a Nikon Ti-E inverted microscope equipped with apochromatic TIRF 60X oil immersion objective lens (N.A. 1.49), pco.edge 4.2 LT sCMOS camera, and SOLA SE II 365 Light Engine as well as complementary DIC components (Nikon Instruments Inc, Melville, NY, USA). Mid-logarithmic phase cells and peptides at the indicated concentration were mixed inside the anaerobic chamber and injected into home-built anaerobic rose-type imaging chambers as previously described [63]. Imaging chambers were removed from the anaerobic chamber and placed on the microscope. The microscope was maintained at 37 °C using a home-built enclosure and a Nevtek Air Stream microscope stage warmer (Nevtek, Williamsville, VA, USA). Nikon Perfect Focus system (Nikon Instruments Inc, Melville, NY, USA) was employed to eliminate focal drift during recordings. Movies consisting of a fluorescence and DIC image each minute for 60 min were then recorded for each condition. Movies started 6–8 min after the bacteria and peptide were mixed. Data analysis was performed using the Nikon Elements imaging suite. During the recordings the amount of fluorescence background increased with time, presumably as peptide was deposited on the coverslip surface. During analysis this background change was corrected using background-leveling tools, then a second rolling-ball type background correction was used to remove imaging artifacts. A thresholded binary mask was then applied to the fluorescence images to isolate and count each fluorescent object (peptide-labeled bacteria) in the movie. Fluorescence levels of objects were monitored as a function of time.

### 4.4. Growth Inhibition Assays

Two-fold dilution series of TY medium containing the indicated concentrations of peptide and/or copper salts were prepared in sterile 96-well plates as detailed in Wiegand et al. [79]. Wells containing 200 μL of medium were inoculated with 20 μL of saturated overnight culture of *C. difficile* 630Δ*erm* or R20291 containing approximately 10^8^ CFU/mL and incubated anaerobically for 16 h at 37 °C. Closed microplates were removed from the anaerobic chamber and the outsides of the plates were disinfected with 10% bleach before examination to determine the minimum concentration of each peptide and/or metal ion sufficient to complete inhibit visible growth. Culture density at 630 nm was measured in a BioTek (Winooski, VT, USA) microplate reader. As removal from the anaerobic chamber killed the anaerobic *C. difficile* bacteria, we were not able to plate samples to determine CFU/mL after spectroscopic measurements. Inhibitory concentrations were reported as the peptide concentration necessary to reduce the overnight OD_630_ by at least 50% from that of untreated samples. Data reported are the means and standard deviations of four biologically independent samples.

### 4.5. Time-Kill Assays

3 mL of TY media were inoculated with single colonies of *C. difficile* R20291 and allowed to grow at 37 °C to an optical density at 600 nm (OD600) of 0.5–0.7. At the onset of the experiment 20 μL aliquots were removed from the exponentially growing culture and inoculated into fresh TY medium containing the indicated concentration of peptide and/or copper sulfate (CuSO_4_). The final volume was adjusted to 1 mL with fresh TY medium. After 0, 0.5, 2, and 4 h of incubation at 37 °C, 10 μL aliquots were removed for serial 10-fold dilution in TY. 10^6^ dilutions were plated in duplicate on BHIS agar plates for colony enumeration. Colony forming units (CFU) were counted after 24 h. Data reported are the averages of three biologically independent samples measured in duplicate. Treated samples were compared to untreated samples and to each other by two-way ANOVA using Tukey’s multiple comparison test with Prism (GraphPad Software, San Diego, CA, USA).

### 4.6. Atomic Absorption Spectroscopy

The copper concentration in TY medium was measured using an AA-7000 atomic absorption spectrophotometer (Shimadzu Scientific Instruments, Columbia, MD, USA) with a hollow cathode lamp using an acetylene flame [80]. Copper from TY medium was detected at 324.8 nm and quantified using a standard curve of copper chloride (CuCl_2_) diluted in water.

## Supplementary Materials

Supplementary materials can be found at https://www.mdpi.com/1422-0067/20/21/5289/s1.
