# Supplementary material for: How Oxygen Availability Affects the Antimicrobial Efficacy of Host Defense Peptides: Lessons Learned from Studying the Copper-Binding Peptides Piscidins 1 and 3
Source: Int J Mol Sci. 2019 Oct 24;20(21):5289. doi: 10.3390/ijms20215289 (PMC6862162; doi:10.3390/ijms20215289)
Supplement: Supplementary file 1 [file ijms-20-05289-s001.pdf]

Supplementary Information

How Oxygen Availability Affects the Antimicrobial Efficacy of Host Defense Peptides: Lessons Learned from Studying the Copper-binding Peptides Piscidins 1 and 3

Adenrele Oludiran[1], David S. Courson[1], Malia D. Stuart[2], Anwar R. Radwan[3], John C. Poutsma[3], Myriam L. Cotten[4*], Erin B. Purcell[1]*

[1] Department of Chemistry and Biochemistry, Old Dominion University, Norfolk, VA, USA

[2] Biology Department, Palomar College, San Marcos, CA, USA

[3] Department of Chemistry, College of William and Mary, Williamsburg, VA, USA

[4] Department of Applied Science, College of William and Mary, Williamsburg, VA, USA

* Corresponding author contact information:

Myriam L. Cotten: mcotten@wm.edu

Erin B. Purcell: epurcell@odu.edu

Table S1: Bacterial Strains

Strain		
Clostridioides difficile 630Δ*erm*	Erythromycin-sensitive derivative of *C. difficile* 630, ribotype 012	Hussain, Roberts, Mullany. J. Med. Micro. 2005.[64]
Clostridioides difficile R20291	2003-2005 UK epidemic strain, ribotype 027	Stabler et al. Genome Biol. 2009.[65]

References cited in Table S1

64. Hussain, H.A.; Roberts, A.P.; Mullany, P. Generation of an erythromycin-sensitive derivative of Clostridium difficile strain 630 (630Deltaerm) and demonstration that the conjugative transposon Tn916DeltaE enters the genome of this strain at multiple sites. *J Med Microbiol* **2005**, *54*, 137-141, doi:10.1099/jmm.0.45790-0.
65. Stabler, R.A.; He, M.; Dawson, L.; Martin, M.; Valiente, E.; Corton, C.; Lawley, T.D.; Sebaihia, M.; Quail, M.A.; Rose, G., et al. Comparative genome and phenotypic analysis of Clostridium difficile 027 strains provides insight into the evolution of a hypervirulent bacterium. *Genome Biol* **2009**, *10*, R102, doi:10.1186/gb-2009-10-9-r102.

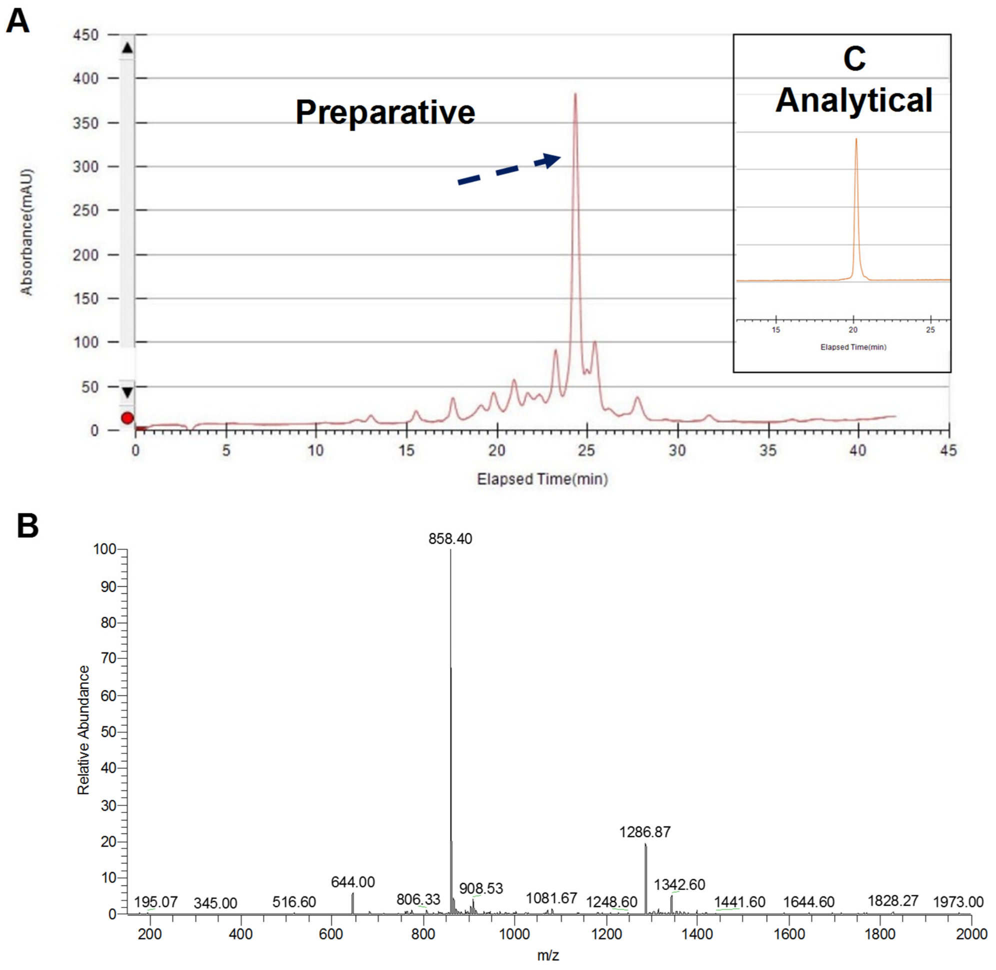

Figure S1: HPLC and mass spectrometry of the P1 peptide. (A) A representative chromatogram of crude P1 is shown. It was obtained on a Waters XBridge C18 preparative column. The HPLC solvents included acetonitrile and water, both acidified with 0.1% trifluoroacetic acid. The method, which was executed at ambient temperature, included running isocratic conditions (85%/15% acetonitrile/water) for 5 minutes, before running a gradient that increased the amount of acetonitrile at a pace of 1.0%/min for 16 minutes, and then 0.50%/min for the following 7 minutes. The main fraction, which eluted at ~32% acetonitrile in the run shown here (blue arrow), was collected and analyzed by mass spectrometry to confirm the isolation of purified P1. (B) P1 was subjected to electrospray ionization mass spectrometry on a Thermo LCQ-DECA instrument. A representative spectrum, which was acquired for ^{15}N-G13-P1 (MW 2572), is shown. It displays the peaks 644.0, 858.4 and 1286.9, corresponding to m/z adducts having z values of +4, +3, and +2, respectively. The small peak detected at 1343 arises from the cationic peptide (z = +2) bound to the anionic trifluoroacetate ion. As indicated in the method, the lyophilized peptide is washed with dilute HCL and dialysis is performed. These steps ensure that the trifluoroacetate ion is removed before performing amino acid analysis (AAA) and biological assays. AAA further confirmed the amino acid content of the peptide and determined the sample concentration. (C) The analytical HPLC chromatogram of a representative P1 sample prepared for AAA is displayed. It was collected on a Waters SunFire C18 analytical column using a gradient similar to that used for the preparative run.

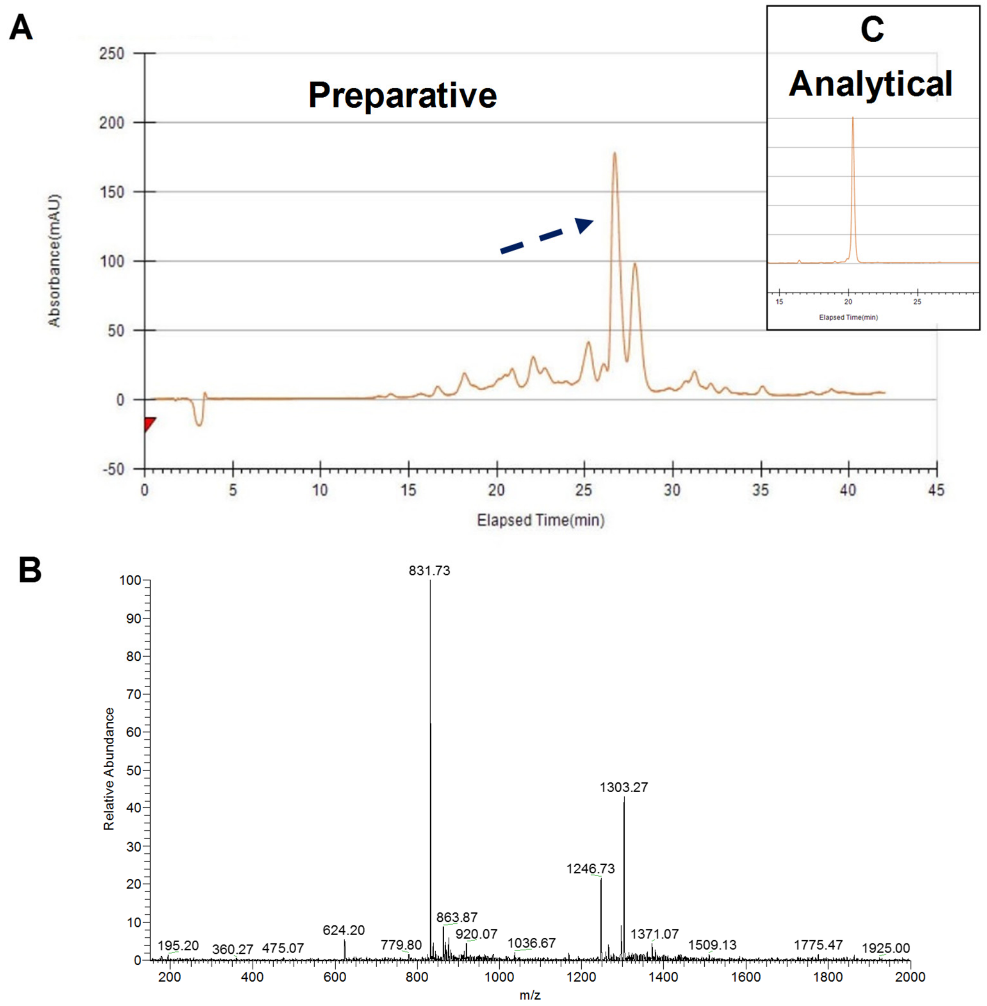

Figure S2: HPLC and mass spectrometry of the P3 peptide. (A) A representative chromatogram of crude P3 is shown. It was obtained on a Waters XBridge C18 preparative column. The HPLC solvents included acetonitrile and water, both acidified with 0.1% trifluoroacetic acid. The method, which was executed at ambient temperature, included running isocratic conditions (85%/15% acetonitrile/water) for 5 minutes, before running a gradient that increased the amount of acetonitrile at a pace of 1.0%/min for 16 minutes, and then 0.50%/min for the following 7 minutes. The main fraction, which eluted at ~33% acetonitrile in the run shown here (blue arrow), was collected and analyzed by mass spectrometry to confirm the isolation of purified P3. (B) P3 was subjected to electrospray ionization mass spectrometry on a Thermo LCQ-DECA instrument. A representative spectrum, which was acquired for ^{15}N-G13-P3 (MW 2492), is shown. It displays the peaks 624.2, 831.7 and 1246.7, corresponding to m/z adducts having z values of +4, +3, and +2, respectively. The peak appearing at 1303 arises from the cationic peptide (z = +2) bound to the anionic trifluoroacetate ion. As indicated in the method, the lyophilized peptide is washed with dilute HCl and dialysis is performed. These steps ensure that the trifluoroacetate ion is removed before performing amino acid analysis (AAA) and biological assays. AAA further confirmed the amino acid content of the peptide and determined the sample concentration. (C) The analytical HPLC chromatogram of a representative P3 sample prepared for AAA is displayed. It was collected on a Waters SunFire C18 analytical column using a gradient similar to that used for the preparative run.
